# Epidemiology of tuberculosis in Chongqing, China: a secular trend from 1992 to 2015

**DOI:** 10.1038/s41598-017-07959-2

**Published:** 2017-08-10

**Authors:** Bo Wu, Ya Yu, Weijia Xie, Ying Liu, Yao Zhang, Daiyu Hu, Yafei Li

**Affiliations:** 1 Department of Epidemiology, College of Preventive Medicine, Third Military Medical University, Chongqing, 400038 People’s Republic of China; 2 Chongqing Institute of Tuberculosis Prevention and Control, Chongqing, 400042 People’s Republic of China

## Abstract

Chongqing is a southwest city of China with high tuberculosis (TB) burden. An observational retrospective study has been performed based on routine TB surveillance data in Chongqing from 1992 to 2015. The TB notification rate has declined to 70.8 cases per 100,000 population from the peak of 106 cases per 100,000 in 2005. The TB notification rate in population over-65 years has become the highest among all-ages population since 2010. The average proportion of farmers in all notified cases from 2008 to 2015 was 62.5%, and the notification rate of farmers has become the highest among all occupations since 2011. The TB notification showed a regional disparity in Chongqing. Despite the improvement achieved since 1992, the TB control efforts has been threatened by new challenges such as the demographic shift towards an aging population, the prevalence of MDR-TB and TB/HIV co-infection, and the regional disparity of TB notification. More effective interventions should be implemented. Our study can serve as a guidance for the future development of TB control in Chongqing, and we believe it has general relevance to TB control in other regions with similar situations.

## Introduction

China is one of the top 22 countries with high tuberculosis (TB) burden, and has the third largest numbers of cases accounting for 10% of the global total^1^. A TB control project based on directly observed treatment, short-course (DOTS) strategy was introduced in 1990s^2^. The prevalence of smear-positive TB was reduced by 65% from 1990 to 2010^3^. The 2015 global TB control goal of reducing the prevalence of smear-positive TB by 50% was reached 5 years ahead of target date in China^4^. The World Health Organization(WHO) has developed the End TB Strategy, with a goal of a 90% reduction in incidence and 95% reduction in mortality by 2035^5^. More effective solution should be established in order to reach the TB targets.

Chongqing is a southwest city of China with 33 million people, comprising 39 districts. Approximately 22,000 TB patients were reported with TB in Chongqing in 2015, making up 2.5% of all notified TB cases in China. The TB case notification rate was 70.8 cases per 100,000 population in Chongqing in 2015, which was higher than the average of the whole country (63.4 cases per 100,000 population). An effective Multi-drug Resistant (MDR) TB control program has been introduced through an international project in Chongqing since 2008. The Center for Disease Control and Prevention (CDC) system and hospital system has cooperated with each other on the treatment of MDR-TB. Human Immunodeficiency Virus (HIV) testing for TB patients has been carried out since 2011.

An observational retrospective study was performed to evaluate the TB prevalence in Chongqing and provided scientific evidence for the future development of TB control strategy. This study has also assessed the effectiveness of the TB control system in Chongqing from 1992 to 2015.

## Methods

### Study design

This was an observational retrospective study of notified TB patients, using TB surveillance data from the national notifiable communicable diseases surveillance system and the national TB surveillance system. The diagnosis of pulmonary TB was based on sputum smear test, and a complete medical evaluation for TB included chest radiology (X-rays), culture, medical history, physical examination, tuberculin skin test and surgical biopsy.

The TB case notification rate from 1992 to 2007 was analyzed, and the effectiveness of the TB control system was mainly measured by the trend of TB case notification rate. The TB patients were stratified by age, sex, occupation, immigration and region for TB notification analysis. The information on sex, age and occupation of TB patients in Chongqing has been reported in the national TB surveillance system since 2008. According to the Chongqing Statistical Yearbook, the population in Chongqing was divided into three age groups: 0–14 years, 15–64 years, and over-65 years. The trends of different age groups were analyzed. The TB patients were divided into farmers and non-farmers to analyze the difference of TB notification. Chongqing comprised 4 regions: the Urban Districts, New Urban Development Districts, Northeast Districts and Southwest Districts, which were defined by local government based on the factors of population, resources, environment, economy, society, culture, etc. The regional disparity of TB notification was evaluated. The imported TB patients were the TB patients from other provinces or other countries. The information of the imported TB patients has been reported in the electronic surveillance system since 2014. The impact of imported TB patients on the local TB prevalence was assessed.

To understand the influence factors for TB notification in Chongqing, we explored potential associated factors including Gross Domestic Product (GDP) per capita of Chongqing, health care expenditure and the proportion of urban population. Limited by local socioeconomic statistical data, the data of this analysis has been extracted since 2007.

The surveillance system for MDR-TB and TB/HIV co-infection has been set up in recent years. The progress in MDR-TB and TB/HIV co-infection was analyzed. According to definitions and reporting framework for tuberculosis of WHO^1^, the treatment success rate, the treatment failure rate, the death rate and the rate of loss to follow-up from 1992 to 2015 were also analyzed. Treatment success was the sum of cured and treatment completed.

### Ethics approval

The study was approved by the Ethics Committee of the Institute of Tuberculosis Prevention and Control, Chongqing, China. As we were carrying out a secondary analysis based on reported data and all individual information was removed before analysis, we were not required to obtain informed consent from individuals.

### Data collection

The data of TB patients from 1992 to 2007 were collected from annual TB reports of Chongqing, and detailed information such as sex, age, occupation and area was not available in this period. The data of TB patients from 2008 to 2015 came from the electronic surveillance system, which provided data for case notifications, diagnosis, treatment, management and the outcomes of TB patients. Reporting of extra-pulmonary TB is not mandatory, thus all data were based on the pulmonary TB patients. Population and socioeconomic data were from Chongqing Statistical Yearbook. The notification data of other countries came from the WHO TB database (http://www.who.int/tb/country/data/download/en/).

### Data Analysis

Comparison between groups was carried out by the chi-square test or Fisher’s exact test. The trend of the notification rate was tested using chi-square test for linear trend. The risk factors of TB notification were analyzed with linear regression. The difference was considered as significant if P-value was less than 0.05. All statistical analyses were performed using SPSS 22.0 (SPSS Inc., Chicago, IL, USA).

### Data Availability

The TB data of Chongqing that support the findings of this study are available from the national surveillance system but restrictions apply to the availability of these data, which were used under license for the current study, and so are not publicly available. Data are however available from the corresponding author upon reasonable request and with permission of the national surveillance system.

## Results

### Trend of TB case notification

**Figure 1:**
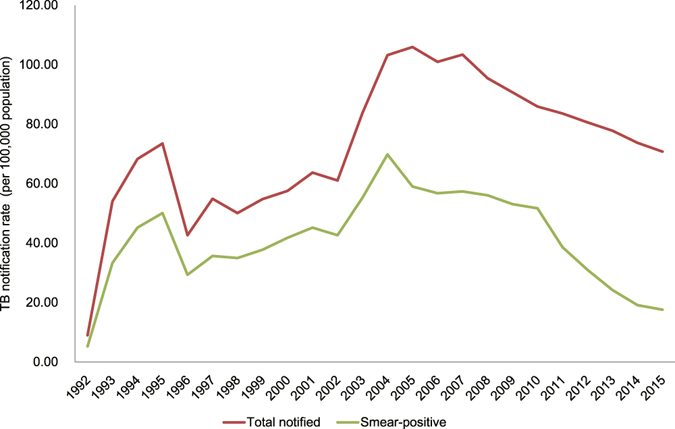
The trend of TB notification rate from 1992 to 2015. The TB notification rate of total notified and smear-positive patients was shown. Total notified TB patients included all forms of TB. Smear-positive TB patients included the new and retreatment patients.

From 1992 to 2015, 472,596 TB cases were notified in Chongqing. Among them, 56% (264,702 TB cases) were smear-positive. The TB case notification rate of all forms increased significantly from 9 cases per 100,000 population in 1992 to a peak of 106 cases per 100,000 population in 2005 (χ^2^ trend = 16,578.5, *P* < 0.05). Since 2005, it has significantly decreased from 106 to 70.8 cases per 100,000 population at an average rate of 3% per year (χ^2^ trend = 4,766.5, *P* < 0.05) (Fig. 1). The notification rate of smear-positive cases showed similar trend, and it has significantly decreased by 74.8% since 2004 (χ^2^ trend = 20,998.1, *P* < 0.05).

### TB case notification stratified by age, sex, occupation and immigration

**Figure 2:**
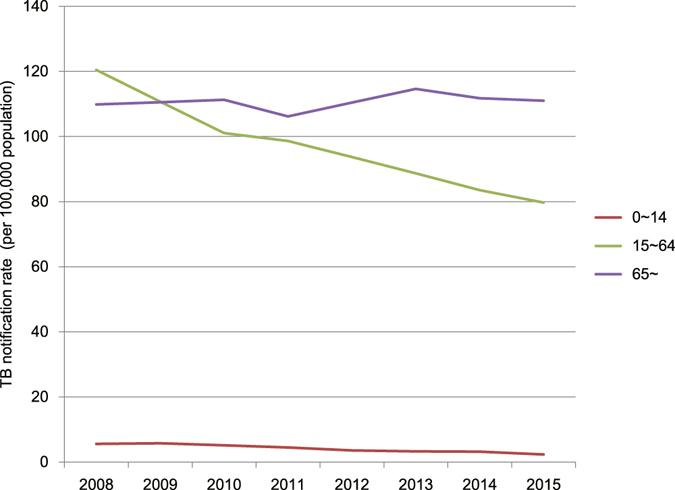
The TB notification rate in different age groups from 2008 to 2015.

The trends of age-specific case notification rates from 2008 to 2015 were different among age groups. The notification rate in the age group 15–64 years had a significant decrease by 33.5% from 120.4 to 80.1 cases per 100,000 population (χ^2^ trend = 2,662.6, *P* < 0.05) (Fig. 2). Children aged 0–14 showed the lowest notification rate, and the notification rate also declined significantly from 5.6 to 2.4 cases per 100,000 population (χ^2^ trend = 125.6, *P* = 3.8 × 10^−29^). The proportion of children aged 0–14 for all forms of TB cases was 0.9% during the period 2008 to 2015. In contrast to the age groups 0–14 and 15–64 years, the notification rate of over-65-year age group did not change significantly (χ^2^ trend = 1.5, *P* = 0.2).

Sex-specific case notification rates of all forms also declined significantly between 2008 to 2015 for both males (χ^2^ trend = 1,131.5, *P* = 4.8 × 10^−248^) and females (χ^2^ trend = 431.1, *P* = 9.2 × 10^−96^). In general, the proportion of male TB patients accounted for 71.3% in this period. There was no significant difference in the sex ratio in all forms of TB patients from 2008 to 2015 (χ^2^ = 12.3, *P* = 0.1).

**Figure 3:**
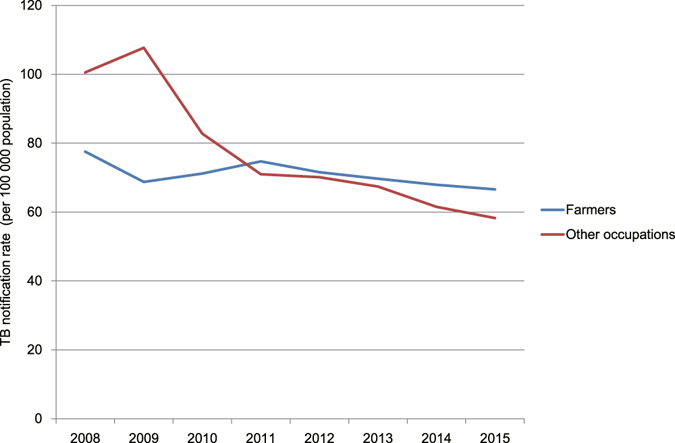
The notification rate of farmers and other occupations from 2008 to 2015.

Most TB patients were farmers, and the average proportion of farmers was 62.5% between 2008 to 2015. The notification rates of farmers (χ^2^ trend = 136.7, *P* = 1.4 × 10^−31^) and all other occupations (χ^2^ trend = 2,757.1, *P* < 0.05) has significantly declined (Fig. 3).

In 2014 and 2015, 370 and 726 imported TB patients were reported in the electronic surveillance system, and the imported TB patients accounted for 0.001% and 0.002% of all TB patients. The notification rate of imported TB patients was 25.3 and 48.3 cases per 100,000 population in 2014 and 2015, respectively.

### Regional disparity of TB notification

**Figure 4:**
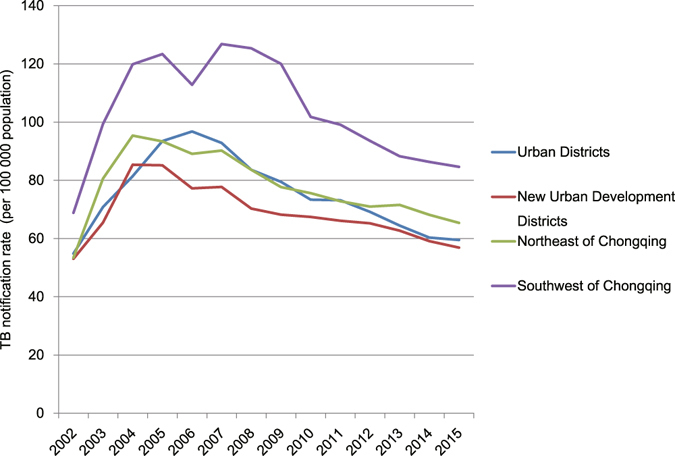
The notification rate of different regions from 2002 to 2015.

The Urban Districts, New Urban Development Districts, Northeast Districts and Southwest Districts showed similar trend of TB case notification rate of all forms from 2002 to 2015. The TB notification rate of Southwest Districts was significantly different from other three regions (χ^2^ = 7,470.6, *P* < 0.05) (Fig. 4).

### TB notification-associated factors

In this analysis, TB notification rate was correlated with GDP per capita of Chongqing (regression coefficient = −0.97, *P* = 1 × 10^−5^), health care expenditure (regression coefficient = −0.95, *P* = 7.8 × 10^−5^), and the proportion of urban population (regression coefficient = −0.99, *P* = 1.5 × 10^−6^).

### MDR-TB and TB/HIV Co-infection

**Figure 5:**
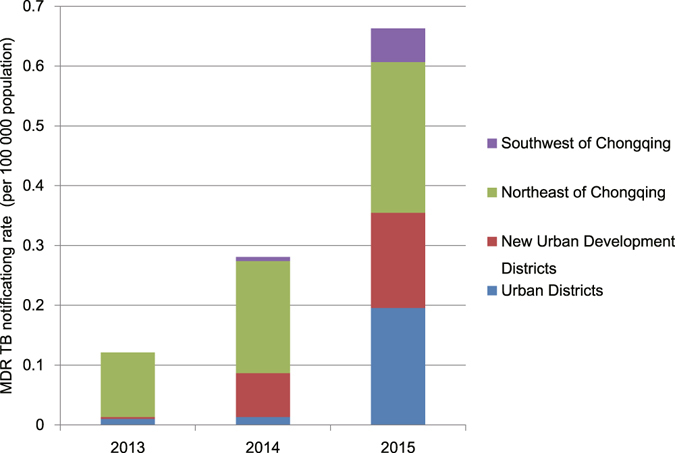
The notification rate of MDR-TB in different regions from 2013 to 2015.

From 2013 to 2015, 320 MDR-TB patients were notified. The MDR-TB case notification rate increased significantly from 0.1 cases per 100,000 population in 2013 to 0.7 cases per 100,000 population in 2015 (χ^2^ trend = 123.5, *P* = 1.1 × 10^−28^) (Fig. 5). The Northeast Districts had the highest MDR-TB case notification rate in all 4 regions (χ^2^ = 130.5, *P* = 4.5 × 10^−29^). There were 19 extensively drug-resistant (XDR) TB patients in the same period, and the average proportion of MDR-TB cases with XDR-TB was 5.9%.

**Figure 6:**
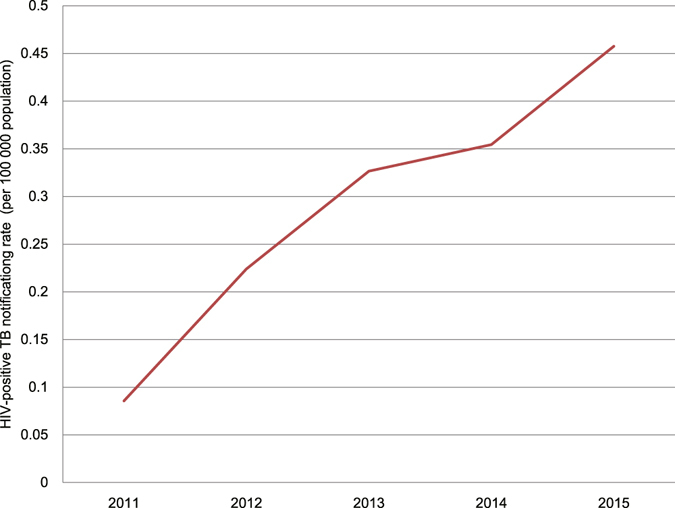
The notification rate of HIV-positive TB patients from 2011 to 2015.

From 2011 to 2015, 46,827 TB patients were tested for HIV, and 432 were confirmed. The notification rate of HIV-positive TB patients increased significantly from 0.086 cases per 100,000 population in 2011 to 0.46 cases per 100,000 population in 2015 (χ^2^ trend = 77.65, *P* = 1.23 × 10^−18^) (Fig. 6). The proportion of TB patients tested for HIV increased significantly from 14.1% in 2011 to 55.7% in 2015 (χ^2^ trend = 8,323.4, *P* < 0.05).

### Treatment Outcome

There were 377,838 new and relapse cases notified from 1992 to 2015, and 358,477 were cured or completed treatment. The treatment success rate of new and relapse cases has been above 90% in this period. The average annual death rate, treatment failure rate and rate of loss to follow-up was 1%, 0.7% and 0.8% respectively from 1992 to 2015.

## Discussion

The TB case notification rate of all forms increased to its peak in 2005, and then decreased at an average rate of 3% per year. Since 2000s, The TB case notification rate of all forms has declined by 33.2% and the notification rate of smear-positive cases has declined by 74.8%. The observed peak of TB notification in 2005 could be explained by several factors. One of the factors was the expansion of the DOTS program to all population since 1992, which provided the free standard short-course chemotherapy in the local CDC system^6^. The DOTS program expansion has greatly improved TB case detection since then. The other factor was the influence of domestic policy. China implemented various policies to help the report and control of TB in 1990s^7^. The TB reporting was mandatory, and the TB patients must be referred from the hospital system to the CDC system for regulatory treatment. The active follow-up was carried out for the referred TB patients who did not get treatment in the CDC system. With these improved measures, more and more TB patients were notified, and the TB case detection rate for new smear-positive increased to 80.7% in 2005. The TB case notification rate of the whole country also reached the point of inflection in 2005^8^.

After 2005, the TB case notification rate has declined. This decrease has benefitted from the intensified public health interventions and the socioeconomic improvement since 2000s. The TB control has been strengthened by increased public-health expenditure, revised laws, the electronic surveillance system, and improved public-health facilities^9^. From 2007 to 2015, the GDP per capita and health care expenditure of Chongqing has increased by 213.9% and 827%, respectively. The increased proportion of urban population has also contributed to it. The TB notification trend of Chongqing is similar with the general trend in China, showing a decrease of 33.2% from 2005 to 2015. While the distinctive decrease has been observed in Chongqing, the TB case notification rate has stabilized or increased in western pacific region and pacific region since 2005^10, 11^. In India, the TB notification rate has increased 24.3% in this period, and it has declined 3.9% in Viet Nam.

Despite the remarkable improvements in TB control, the TB case notification rate is higher in the western region of China than in the eastern and central regions, and Chongqing is one of the high TB burden cities in China^8^. The national surveys also showed that the west region had the highest TB prevalence, and the decrease in prevalence of bacteriologically positive TB was lowest compared with the eastern and central regions^3, 12, 13^. Furthermore, analysis in our study showed a regional disparity in the TB notification rate within the city. The TB notification rate of Southwest Districts was the highest in all 4 regions. This regional disparity may result from the difference in socioeconomic development. The Southwest Districts had the lowest socioeconomic status. In our study, the GDP per capita, health care expenditure per capita and the proportion of urban population have significant influence to TB notification. Similar significant correlation was also shown in other studies^14–16^. Increased financial and policy support should be needed to improve TB control efforts in the western under-developed regions.

The case notification rate of over-65-year age group has not decreased from 2008 to 2015, and it became the highest in all age groups. The aging population of Chongqing is expanding fast. The proportion of over-65-year age group population has increased from 8.8% to 12.2% since 2000. The control of TB in elderly people has become a great challenge. The high case notification rate of elderly people might be due to various risk factors, like age-related immune dysfunction^17^, socioeconomic status, diabetes^18, 19^ and chronic respiratory diseases^20^. More effective interventions including active case detection and easy access to high-quality health care for the elder should be implemented. Such efforts will contribute to the future reduction in the TB prevalence^21^.

Men are more likely to develop TB than women. The proportion of male TB patients accounted for 71.3% from 2008 to 2015 in Chongqing. This gender difference has also been reported in the China national surveys^3^ and in other countries and regions^10, 22^. The gender difference may come from biological differences like steroid hormones and genetic variants^23–25^.

The notification rate of farmers has gone beyond that of all other occupations since 2011. TB was closely associated with poverty^26^. Many farmers lived in underdeveloped regions of Chongqing, and the annual income of farmers was roughly 40% of urban residents according to Chongqing Statistical Yearbook in recent years. The low socioeconomic status of farmers may cause the higher notification rate of farmers.

The proportion of imported TB patients was very low, and the notification rate of imported TB patients was lower than that of all forms. The impact of imported TB patients on the local TB prevalence was limited.

The proportion of notified MDR-TB cases in smear-positive TB patients was 3.8% in 2015. In 2007 and 2010 two national surveys about MDR-TB were carried out in China, and reported 5.7% and 6.8% MDR-TB among the smear-positive patients, respectively^3, 27^. The MDR-TB proportion of Chongqing in 2015 was lower than that in the previous national surveys. This result may suggest that the under-notification of MDR-TB has occurred in Chongqing. By now, the screening has been carried out in high risk group of MDR-TB, including retreatment, failure, close contacts of MDR-TB, and smear-positive after 2–3 months of treatment. To improve the notification of MDR-TB, the screening range should be widened. From 2017, 20% of new smear-positive patients will be screened for MDR-TB in Chongqing. Furthermore, the rapid molecular test like Xpert MTB/RIF test has been introduced in recent years^28, 29^. That has also contributed to the notification of MDR-TB. Predictably, more MDR-TB patients will be found out with development of active case-detection in the future.

The prevalence of HIV/TB co-infection is indeed worth more attention^1, 30^. WHO recommended that routine HIV testing should be offered to all TB patients^31^. The proportion of positive patients in HIV test has had a steady growth from 0.7% to 1.2% since 2011 in Chongqing. According to this growth rate, we will face a increasing challenge for the TB/HIV control in the near future. Strengthening cooperation and coordination between the control of TB and HIV/AIDS should be put on the agenda as soon as possible.

TB control over the years has been effective in Chongqing, and the TB case notification rate has declined after 2005. For further reduction in tuberculosis prevalence, new challenges should be focused on. In Chongqing, growing challenges to TB control have been posed by a regional disparity due to socioeconomic status, the high TB case notification rate of elderly people, the under-notification of MDR-TB, and the increasing prevalence of TB/HIV co-infection. More effective interventions will likely be needed, including increasing financial and policy support in the western under-developed regions, active case detection for the elder, widening the screening range of MDR-TB, strengthening the control of TB and HIV/AIDS.

This study has limitations. The epidemiological survey for TB has not been conducted in Chongqing, so the routine TB surveillance data has been analyzed in our study. The data of TB patients before 2008 were from annual TB reports of Chongqing, and detailed information like sex, age, occupation and area is not available. Demographic data from Chongqing Statistical Yearbook did not show detailed age information, and the population could only be divided into three age groups: 0–14 years, 15–64 years, and over-65 years. In some data analyses, the actual P-value could not be generated by SPSS when P-value is too small to be showed.
